# Cathepsin D elevates the fibrocalcific activity in human aortic valve cells through the ERK1/2-Sox9 pathway

**DOI:** 10.3389/fcvm.2024.1410862

**Published:** 2024-09-24

**Authors:** Qingzhou Yao, Erlinda The, Balachandar Nedumaran, Yufeng Zhai, Lihua Ao, David A. Fullerton, Xianzhong Meng

**Affiliations:** Department of Surgery, University of Colorado, Denver, CO, United States

**Keywords:** cathepsin D, matrilin 2, Sox9, fibrocalcification, CAVD

## Abstract

**Background:**

Human Aortic valve interstitial cells (AVICs) from calcific aortic valve disease (CAVD)-affected valves exhibit elevated fibrocalcific activity although the underlying mechanism remains incompletely understood. This study aimed to identify endogenous factors that promote aortic valve fibrocalcification.

**Methods and results:**

Proteomic analysis found increased cathepsin D levels in AVICs from CAVD-affected valves compared to AVICs from normal valves, and this finding was validated by immunoblotting. ELISA assay identified exacerbated release of cathepsin D by AVICs of diseased valves. Recombinant human cathepsin D upregulated the expression of runt-related transcription factor 2 (Runx2), alkaline phosphatase (ALP), collagen I and collagen IV in human AVICs, resulting in the deposition of calcium and collagen. Blocking of the ERK1/2-Sox9 signaling pathway markedly reduced the pro-fibrocalcific effect of cathepsin D. Moreover, normal AVICs express and release greater levels of cathepsin D when exposed to soluble matrilin 2. Knockdown of cathepsin D attenuated the fibrocalcific response induced by soluble matrilin 2.

**Conclusion:**

AVICs of diseased aortic valves produce and release greater levels of cathepsin D that exerts a pro-fibrocalcific effect on AVICs through the ERK1/2-Sox9 pathway. Soluble matrilin 2 up-regulates cathepsin D to elevate AVIC fibrocalcific activity. Over-expression of cathepsin D in the aortic valve may enhance the pathobiological activities in AVICs.

## 1 Introduction

Calcific aortic valve disease (CAVD) is common in people over 65 years of age and is becoming a major healthcare issue worldwide (1, 2). As there is no pharmaceutical intervention, CAVD frequently progresses to aortic valve stenosis that requires surgical or transcatheter replacement of the aortic valve. However, complications due to anti-coagulation and the short lifespan of prosthetic valves are the limitations of valve re-placement treatment. In addition, advanced age, and aging-related conditions in CAVD patients may increase the risk for complications associated with valve replacement. Therefore, understanding the mechanisms of CAVD pathogenesis and identifying therapeutic targets to prevent its progression are of great significance.

CAVD pathobiology is characterized by progressive fibrosis and calcification in the aortic valve leaflets. Previous studies have shown that aortic valve interstitial cells (AVICs) in diseased valves differentiate into myofibroblasts and osteoblast-like cells, exhibiting increased collagen deposition and over-expression of osteogenic factors alkaline phosphatase (ALP) and runt-related transcription factor 2 (Runx2) (3). When activated by pathological factors such as pro-inflammatory cytokines, mechanical stress, and changes in extracellular matrix (ECM) composition, AVICs can transform into myofibroblasts or osteoblast-like cell (3). It is reported that myofibroblast-like AVICs augment ECM remodeling and promote collagen deposition in the valvular leaflets, while osteoblast-like AVICs are responsible for active calcium deposition (4). These AVIC phenotypic changes are believed to be critical for CAVD progression. As such, valvular myofibroblastic-like and osteoblast-like cells have emerged as targets for pharmacological intervention. However, the factors that drive phenotypic changes in human AVICs remain to be identified.

Several studies analyzed gene expression in aortic valves affected by CAVD and provided useful information for improving the understanding of CAVD-associated genetic alterations (4, 5). While proteomics can identify alterations in protein levels and provide insight into the molecular mechanism underlying CAVD pathobiology, limited numbers of studies have been performed using the proteomic approach (6–8). Previous proteomic analyses compared the protein profiles of diseased aortic valve tissue and normal aortic valve tissue and found that calcified aortic valves display many alterations in protein levels when compared to normal valves (7, 9). The limitations of analyzing aortic valve tissue include the contribution by multiple cell types, including inflammatory infiltrates, myofibroblasts and osteoblast-like cells that are present in diseased aortic valves. A recent study analyzed AVICs isolated from CAVD donors using the proteomic approach and identified a disease-driver population (10). This work advanced the understating of CAVD pathogenesis. Further work is needed to identify the unique protein profile in AVICs of diseased aortic valves by contrasting to AVICs of normal aortic valves. Such studies may identify novel molecules that mediate AVIC fibrogenic and osteogenic activities critical for CAVD progression.

Using microRNA array analysis of AVICs from normal aortic valves and diseased aortic valves, our previous studies found the alteration in microRNA expression in diseased human aortic valves and confirmed the role of miR-204 insufficiency and miR-486 over-expression in AVIC fibrocalcific activation associated with CAVD progression (11, 12). Similarly, our gene array analysis of AVICs from normal aortic valves and diseased aortic valves identified down-regulated ADAMTS5 gene expression in AVICs of diseased valves (13). Further, matrilin 2 accumulation associated with ADAMTS5 insufficiency in diseased aortic valves plays a role in elevation of AVIC fibrocalcific activity (13). Matrilin 2 can be released in a soluble form from the extracellular matrix or by cells during tissue stress or injury. We have reported that soluble matrilin 2 exerts a pro-inflammatory effect on AVICs through Toll-like receptor (TLR) 2 and 4 (14), and that soluble matrilin 2 up-regulates the pro-osteogenic factor ALP in human AVICs (13). Therefore, systemic analysis of AVICs from diseased valves may identify novel factors involved in the mechanism underlying AVIC fibrocalcific activation.

The main purpose of the present study was to identify the alteration of levels of proteins that may modulate AVIC fibrocalcific activity. Specifically, we applied proteomic analysis to identify candidate protein and evaluated its effect on AVIC fibrocalcific activity.

## 2 Materials and methods

### 2.1 Isolation and culture of AVICs

This study complied with the Declaration of Helsinki and was approved by the Institutional Review Board of the University of Colorado. All patients provided written informed consent for the use of their aortic valves for this study. As shown in Supplementary Table 1, calcified aortic valve leaflets were obtained intraoperatively from patients undergoing aortic valve replacement at the University of Colorado Hospital due to aortic stenosis (4 males and 1 female; age 59.6 ± 5.1 years). Normal aortic valves were collected from explanted hearts of patients who had cardiomyopathy and were undergoing heart transplantation at the University of Colorado Hospital (3 males and 2 females; age 57.8 ± 3.8 years). The valve leaflets from explanted hearts from patients without heart valve disease were thin and had no histological abnormality.

AVICs were isolated and cultured using a previously described method (15). Briefly, valve leaflets were subjected to initial digestion with a higher concentration of collagenase (2.5 mg/ml) to remove endothelial cells. Then the remaining tissue was treated with a lower concentration of collagenase (0.8 mg/ml) to free interstitial cells. Interstitial cells were collected by centrifugation and cultured in M199 growth medium (Lonza) containing penicillin G, streptomycin, amphotericin B and 10% fetal bovine serum (Thermo Fisher Scientific.Inc) AVIC isolates obtained using this modified protocol lack endothelial cells as verified by negative von Willebrand factor staining (15). One isolate was obtained from each donor aortic valve and was used as an independent sample. Cells of passage 3–6 were used for the experiments.

When cells grew to 80%–90% confluence, cell lysate protein samples from 5 normal and 5 diseased AVIC isolates were collected for proteomic analysis. Cells lysate and culture medium from normal and diseased AVICs were collected to determine the level of cathepsin D using immunoblotting and ELISA kit.

To detect pro-form cathepsin D in the cell culture medium through immunoblotting, the cell culture medium was collected and concentrated using a centrifugal filter device (Milli-poreSigma). The medium was concentrated 60×. Subsequently, the total protein levels in the concentrated medium were determined by measuring the absorbance at 280 nm (UV range). Immunoblotting was performed to detect both the concentrated cell culture medium and the corresponding cell lysate.

AVICs from normal valves were stimulated with recombinant human (rh) cathepsin D (R&D Systems) of varying concentrations for different periods to assess its effect on cellular fibrogenic activity, osteogenic activity, and ERK1/2 activation. AVICs from normal valves were stimulated with matrilin 2 (2 µg/ml, R&D system) to examine cathepsin D expression using immunoblotting and ELISA.

To inhibit ERK1/2, cells were pretreated with ERK1/2 inhibitor PD98059 (25 µmol/L, Selleckchem) 1 h before being exposed to rh-cathepsin D. Immunfluorescence for Sox9 was performed in AVICs corresponding to rh-cathepsin D and ERK1/2 inhibitor incubation experiments.

To knockdown cathepsin D or Sox9, cells (60%–70% confluent) on 24-well plates were incubated were treated with shRNA (constructed from the Functional Genomics Facility of the University of Colorado) before the addition of matrilin 2 or rh-cathepsin D. Recombinant lentivirus expressing control shRNA (CCGGCAACAAGATGAAGAGAGCACAACTCGATGGTGCTCTTCATCTTGTTGTTTT, 100 nmol/L), Sox9 shRNA (target sequence: ACTTCGATGTCAACGAGTTT, 100 mmol/L, or cathepsin D shRNA (target sequence: CATCACCTTCATCGCAGCCAA, 100 nmol/L) in the presence of puromycin (5 µg/ml). Three days later, cells were harvested for validation of knockdown or stimulated with matrilin 2 or rh-cathepsin D for 48 h to determine the role of Sox9 or cathepsin D in the up-regulation of cellular fibrocalcific activities.

All experiments were repeated using cells isolated from different donor valves. Experiments were performed using triplicate wells for each treatment of cells from the same isolate. To ensure equal amounts of cells were used in the experiments, we performed cell counting and seeded equal number of cells to each well to start the experiments. In addition, we checked cell density in the wells at the end of the experiments to confirm that no significant difference in cell density exists among the wells assigned to different treatments.

### 2.2 Proteomic and mass spectrometry analysis

AVIC protein samples were processed using a previously described method. Briefly, AVICs samples from 5 normal and 5 diseased aortic valves were collected by solubilizing cells in lysis buffer (0.01 m Tris-HCl, pH 7.4, 0.14 m NaCl, 1% Triton X-100, and a protease inhibitor mixture (Sigma-Aldrich Chemical Co). The lysate was centrifuged at 4°C for 40 min and supernatants were collected and measured for protein concentration by BCA assay following the manufacturer's protocol (Sigma-Aldrich Chemical Co). Aliquots of 50 μg of protein were made from each sample and subsequently precipitated with 10 mM NaCl and ice-cold acetone. Samples were centrifuged at 18,000 xg for 10 min at 4°C to pellet protein and then supernatant was removed.

Protein pellets were digested using the Preomics iST sample preparation kit (Preomics GmbH, Planegg/Martinsried, Germany) according to the manufacturers protocol. Briefly, Protein Pellets were solubilized in lysis buffer and then heated at 95°C for 10 min to reduce and alkylate proteins. Samples were then sonicated for 10 min and then tryptically digested for 3 h at 37°C on a shaker at 500 rpm. Digestion was stopped and then samples were cleaned up using preomics cartridges and wash and elution buffers. Samples were dried down via SpeedVac and re-suspended in LC-Load buffer provided by preomics iST kit at a concentration of 0.2 µg/µl. Normal samples were then pooled, and disease samples were pooled and analyzed by LC MS/MS. Digested pooled samples were analyzed on a Bruker Maxis Impact HD QTOF mass spectrometer equipped with a CaptiveSpray ion source and a nano-Advance HPLC system (Bruker, Bilerica, MA). Samples were loaded onto a Capillary ProntoSIL C18AQ Trapping column and chromatographically resolved online using a ProtntoSIL C18AQ 100 µM × 150 mm (3.0 u, 200 A) analytical column from NanoLCMS Solutions. Mobile Phases consisted of water in 0.1% formic acid (mobile phase A) and 90% aqueous acetonitrile in 0.1% formic acid (mobile phase B). Samples were loaded onto the trapping column at 5.0 μl/min for 4 min at initial condition before being chromatographically separated at a flow rate of 0.5 μl/min using a gradient of 3%–8% B over 5 min, 8%–40% B over 55 min for a total 60 min gradient at 40⁰C. The column was then washed at 90% B for 5 min before being re-equilibrated at initial condition. MS/MS data was collected by Data Dependent Acquisition. The mass spectrometry experiments were performed at the University of Colorado Proteomics and Mass Spectrometry Facility.

### 2.3 Ms data processing and bioinformatic analysis

MS/MS spectra were extracted from raw data files and converted into mgf files using Bruker Data Analysis software. MGF files were then searched using ProteinScape software (Bruker, ver. 3.1) with Mascot search engine (version 2.6, Matrix Sciences). Data was searched against SwissProt Homo sapiens database using semi-specific trypsin enzyme allowing 2 missed tryptic cleavages with variable carbamidomethyl (C), deamidated (NQ), and oxidation (M) modifications (16). An FDR of 1% was used as a cut-off for peptide identification. Identified peptide areas were rolled up into protein level data and fold change calculations were performed between pooled normal and diseased samples. Data was assessed qualitatively via fold change calculations.

### 2.4 Immunofluorescence staining and confocal microscopy

AVICs were seeded on 8-well chamber slides and treated with or without rh-cathepsin D (40 nM) for the indicated periods. If needed, the specific ERK1/2 inhibitor (PD98059, 25 μM) was added 1 h before the addition of rh-cathepsin D. Immunofluorescence staining was performed as previously described to localize Sox9 (17). After permeabilization with a methanol/acetone mixture, cells were fixed in 4% paraformaldehyde and then incubated with primary antibodies against Sox9 (Cell Signaling, Inc; 1:100 dilution) overnight at 4°C. After washing with PBS, cells were incubated with a Cy3-tagged secondary antibody (imaged on red channel, Thermo Fisher Scientific.Inc). 4', 6-diamidino-2-phenylindole (DAPI, Sigma-Aldrich Chemical Co) was used for nucleus counterstaining (imaged on the blue channel). Microscopy was performed with a Leica CTR5500 digital microscope (Leica Mikroskopie and Systeme GmbH, Wetzlar, Germany). Imagine J software (version 1.53) was applied to determine Sox9 protein density in the nuclei. The “Analyze Particles” function enabled selection and measurement of the areas covered by nuclear dye and obtain integrated density value that represents average amount of Sox9 signal within a nucleus. Nuclear Sox9 density normalized by area was derived by dividing the integrated density by the area of each nucleus.

### 2.5 Immunoblotting

Immunoblotting was applied to analyze cathepsin D (Cell Signaling, Inc, E5V4H, Cat. #74089), Runx2 (Cell Signaling, Inc, D1L7F, Cat. #12556), ALP (Abcam, EPR27191-14, Cat. ab307726), Sox9 (Cell Signaling, Inc, D8G8H, #82630), collagen I (Abcam, EPR22894, Cat. ab6308), collagen IV (Abcam, EPR20966, Cat. ab6586), β-actin (Abcam, SP124, Cat. ab8227), phosphorylated (Cell Signaling, Inc, Thr202/Tyr204, D13.14.4E, Cat. #4370) and total ERK1/2 (Cell Signaling, Inc, 137F5, Cat. #4695) and GAPDH (Cell Signaling, Inc, D16H11, Cat. #5174). Cells were lysed in a commercial sample buffer according to the manufacturer's instructions. The protein samples were resolved on 4%–20% SDS-PAGE gels and then transferred onto nitrocellulose membranes using a wet transfer system. After blocking with 5% skim milk at room temperature for 1 h, membranes were incubated with primary antibodies at 4°C overnight, followed by incubation with appropriate horseradish peroxidase (HRP)-conjugated secondary antibody (Thermo Fisher Scientific, Inc) in 5% skim milk for 1 h. Protein bands were detected using the enhanced chemiluminescence system. Band density was analyzed using the National Institutes of Health Image J software (Wayne Rasband, National Institutes of Health, Bethesda, MD). The fold change of the target bands was normalized using the corresponding internal loading protein, which was either GAPDH or β-actin. This normalization procedure allows for accurate quantification and comparison of the target protein levels across different samples. The data presented in the study represent the mean value ± standard error (SE) derived from multiple independent experiments using different cell isolates obtained from distinct valves.

### 2.6 Alizarin red S staining

For analysis of calcium deposition, AVICs were seeded on 24-well plates. When reaching 80%–90% confluence, cells were treated with indicated interventions in a conditioning medium (growth medium supplemented with 10 mmol/L β-glycerophosphate, 10 nmol/L dexamethasone, 4 μg/ml cholecalciferol and 4 mmol/L CaCl_2,_ Sigma-Aldrich Chemical Co) for 10 days. The medium was changed every 3 days during this period. At the end of the experiments, alizarin red staining and quantification of calcium deposition were performed as previously described (18).

### 2.7 Picro-sirius red (PSR) staining

PSR staining speciﬁcally identiﬁes collagens and is a useful method for the assessment of ﬁbrogenic activity in cultured cells (19). Human AVICs were treated with the indicated intervention in normal cell culture medium for 14 days. Cells were fixed with methanol (Sigma-Aldrich Chemical Co) overnight at −20°C. Following washes with PBS, the cells were incubated in 0.1% PSR (Sigma-Aldrich Chemical Co) for 4 h. After rinses with 0.1% acetic acid (Sigma-Aldrich Chemical Co) plates were air-dried and examined using microscopy. For the quantiﬁcation of PSR staining, stained cells were treated with 100 µl of 0.1 M sodium hydroxide (Sigma-Aldrich Chemical Co) for 2 h to elute the stain. Optical density was determined using a spectrophotometer (BioTek Instruments) at 540 nm.

### 2.8 Enzyme-linked immunosorbent assay (ELISA)

Diseased or Normal AVICs were cultured on the plate with or without treatment for 48 h. Cell culture supernatants were collected. Level of cathepsin D was analyzed in triplicates using an ELISA kit (R&D System) following the manufacturer's protocol. Absorbance of standards and samples were determined spectrophotometrically at 450 nm, using a microplate reader (Bio-Rad Laboratories, Inc, Hercules, CA). Results were plotted against the linear portion of a standard curve.

### 2.9 Statistical analysis

Statistical analysis was performed using the Statistical Package for Social Sciences, version 17.0 (SPSS Inc, Chicago, IL, USA). Data are presented as mean ± standard error (SE). After confirming that all variables were normally distributed using the Kolmogorov-Smirnov test, student *t*-test was applied for comparisons between two groups, and one-way ANOVA was used to analyze differences between multiple groups. *P* < 0.05 was accepted as statistically significant.

## 3 Results

### 3.1 Diseased AVICs express and release higher levels of cathepsin D

**Figure 1:**
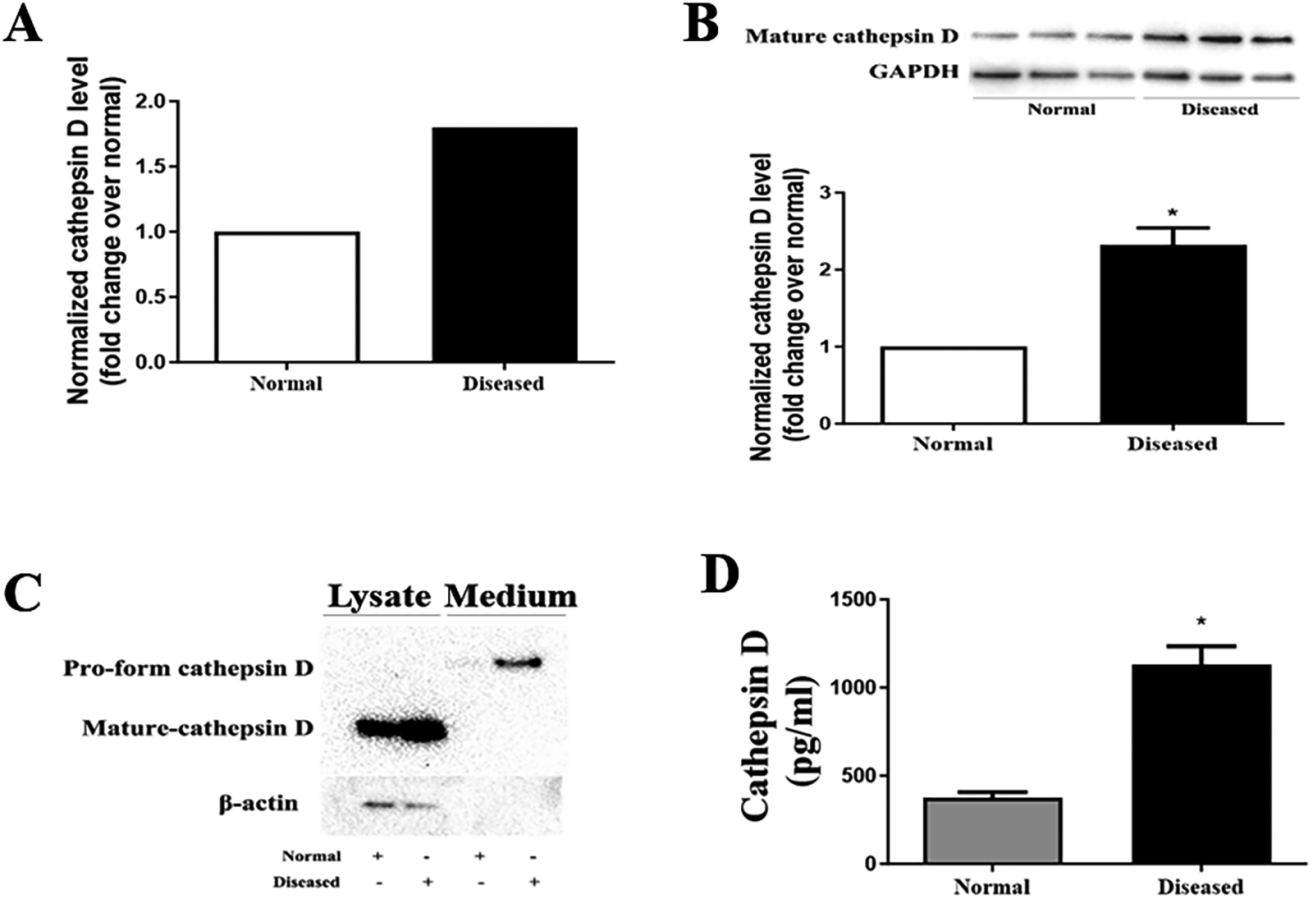
AVICs of diseased valves express and release greater levels of cathepsin D. **(A)** Cell lysates from 5 normal and 5 diseased aortic valves were collected to conduct proteomic analysis. As revealed by proteomic analysis data, cathepsin D protein level is 1.8-fold higher in AVICs from aortic valves affected by CAVD compared to that in cells from normal valves. **(B)** AVICs from normal and diseased valves were cultured for 48 h without treatment, immunoblotting was applied to examine cathepsin D levels in cell lysate. Representative immunoblots and densitometric data show that diseased AVICs have higher levels of cell-associated cathepsin D. All data are presented as mean ± SE of 5 experiments using cell isolates from distinct valves. *P < 0.05 vs. normal. **(C)** AVICs were cultured for 48 h, and the supernatant and cell lysate were collected. Concentrated medium (60×) and cell lysates were analyzed by immunoblotting. Representative immunoblots and densitometric data show that diseased AVICs release greater levels of pro-form cathepsin D into cell culture medium and also have higher levels of cell-associated cathepsin D. **(D)** AVICs from normal and diseased valves were cultured for 48 h, and the medium was collected. ELISA was applied to examine the level of pro-form cathepsin D. Higher levels of pro-form cathepsin D were detected in cell culture medium of diseased AVICs. All data are presented as mean ± SE of 5 experiments using cell isolates from distinct valves. *P < 0.05 vs. normal.

Proteomic analysis was applied to characterize the levels of proteins in AVICs from normal and diseased aortic valves. We pooled the protein samples from 5 AVIC isolates in each group. The proteomic analysis detected 1,304 proteins in human AVICs from diseased valves and 1,099 proteins in human AVICs from normal valves. Of these, 964 proteins were detected in both cell phenotypes. With a fold change cutoff of 1.5, we identified 7 down-regulated proteins and 64 up-regulated proteins in AVICs from diseased valves, as listed in Supplementary Table 2. Notably, a 1.8-fold increase in cathepsin D level was observed in AVICs from diseased valves (Figure 1A). Subsequent bioinformatic analysis and literature review highlighted the significant association of cathepsin D with cardiovascular diseases and the progression of fibrogenesis. Consequently, we conducted immunoblotting and ELISA experiments to validate the expression of cathepsin D in human AVICs.

Cathepsin D is synthesized as an inactive preprocathepsin D that is cleaved and glycosylated to form procathepsin D and then further cleaved to produce heavy and light chains, respectively (20). To confirm the proteomic results, we examined cathepsin D levels in cell lysate from normal and diseased AVICs using immunoblotting. The antibody we used specifically recognizes preprocathepsin D (43 kDa), procathepsin D (46 kDa), and the heavy chain subunit of mature cathepsin D (28 kDa). It is reported that cell-associated cathepsin D is mainly in the mature form (in lysosomes), and the pro-form of cathepsin D is the main form secreted from the cytoplasm. We observed significantly higher levels of the mature form (heavy chain subunit of the mature form) of cathepsin D in the diseased AVICs (Figure 1B). However, the bands of procathepsin D in both normal and diseased AVICs samples were faint. As the pro-form cathepsin D is the main form secreted, we performed immunoblotting to detect this form of cathepsin D in the concentrated (60×) cell culture medium. Due to the unavailability of control antibodies in the cell culture medium, we collected the retained cell lysate for further analysis. To ensure accurate normalization of protein levels within each group, we employed β-actin as a loading control. This approach helps account for potential variations in protein expression levels due to differences in the number of cells between groups. By using β-actin as a reference protein, we partially reflect the relative cell numbers in each group, allowing for more reliable comparisons of protein expression levels. The results in Figure 1C show that diseased AVICs release higher levels of procathepsin D (46 kDa) and also have greater levels of cell-associated mature cathepsin D. To confirm the findings that diseased AVICs release greater levels of cathepsin D, we applied ELISA to determine the levels of cathepsin D in unconcentrated medium from cultures of normal and diseased AVICs. The result in Figure 1D shows that diseased AVICs release significantly higher levels of pro-form cathepsin D (1,129 ± 181.8 pg/ml) compared to normal AVICs (375.5 ± 55.04 pg/ml).

### 3.2 Cathepsin D elevates the osteogenic and fibrogenic activities in human AVICs

**Figure 2:**
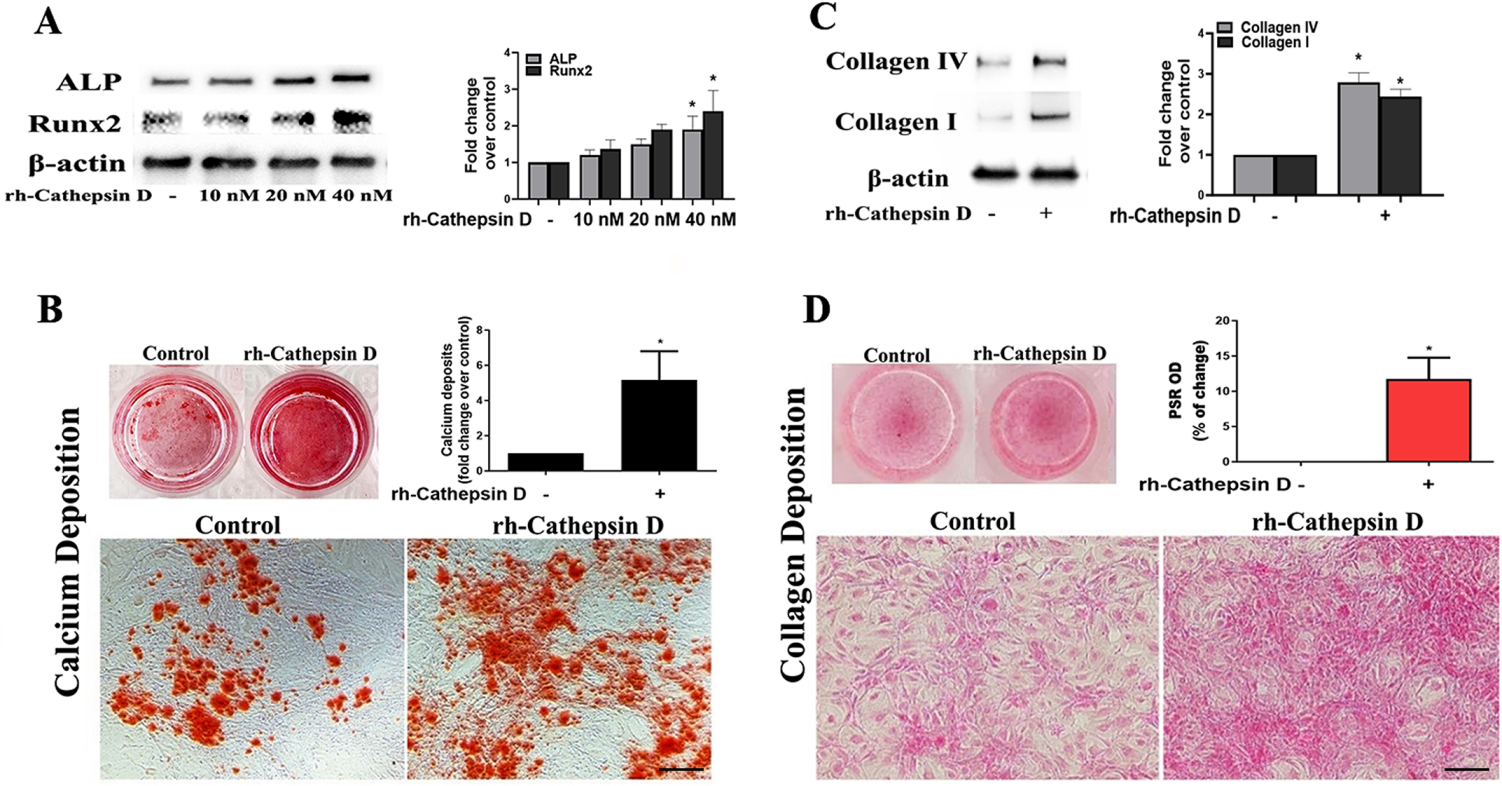
Cathepsin D elevates fibrocalcific activity in human AVICs. **(A)** Human AVICs from 5 normal valves were treated in triplicates with recombinant human cathepsin D (rh-cathepsin D, 10–40 nM) for 48 h. Representative immunoblots and densitometric data show that rh-cathepsin D up-regulates ALP and Runx2 levels in a dose-dependent manner. **(B)** Human AVICs from normal valves were treated with rh-cathepsin D (40 nM) for 10 days in a conditioning medium. Representative images of alizarin red staining (10× objective for original magnification of the higher power images, scale bar = 100 µm) and spectrophotometric data show that rh-cathepsin D promotes calcium deposit formation. **(C)** Human AVICs from normal valves were treated with rh-cathepsin D (40 nM) for 48 h. Representative immunoblots and densitometric data show that cathepsin D up-regulates collagen IV and collagen I levels. **(D)** Human AVICs were treated with rh-cathepsin D (40 nM) for 14 days. Representative images of PSR staining (10× objective for original magnification of the higher power images, scale bar = 100 µm) and spectrophotometric data show that cathepsin D promotes collagen deposition. All data are presented as mean ± SE of 5 experiments using cell isolates from distinct valves. *P < 0.05 vs. control.

To determine the effect of cathepsin D on human AVICs, we treated normal cells with rh-cathepsin D (10–40 nM) for 48 h. The rh-cathepsin D is a pro-form protein with a molecular size of 44 kDa. We observed that rh-cathepsin D markedly up-regulated ALP and Runx2 levels at the dose of 40 nM (Figure 2A). When cells were treated with this dose of rh-cathepsin D for 10 days in a conditioning medium, they produced greater amounts of calcium deposits (Figure 2B). CCK-8 test confirmed that cathepsin D has no effect on cell viability (Supplementary Figure 1). Thus, the observed calcification following rh-cathepsin D treatment is not induced by cell death.

Treatment with rh-cathepsin D up-regulated the expression of collagen I and collagen IV (Figure 2C) and promoted collagen deposition when the treatment was prolonged (Figure 2D). In the aortic valve, collagen I is the predominant collagen type, while collagen IV is a significant constituent of the basement membrane. Both collagen I and collagen IV play crucial roles in providing structural support and preserving the integrity of the valve tissue. Changes in the levels of collagen I and collagen IV serve as indicators of fibrogenic alterations occurring in aortic valve. Therefore, cathepsin D is capable of up-regulating osteogenic and fibrogenic activities in human AVICs.

### 3.3 Cathepsin D activates the ERK1/2-Sox9 pathway to elevate the osteogenic and fibrogenic activities in human AVICs

**Figure 3:**
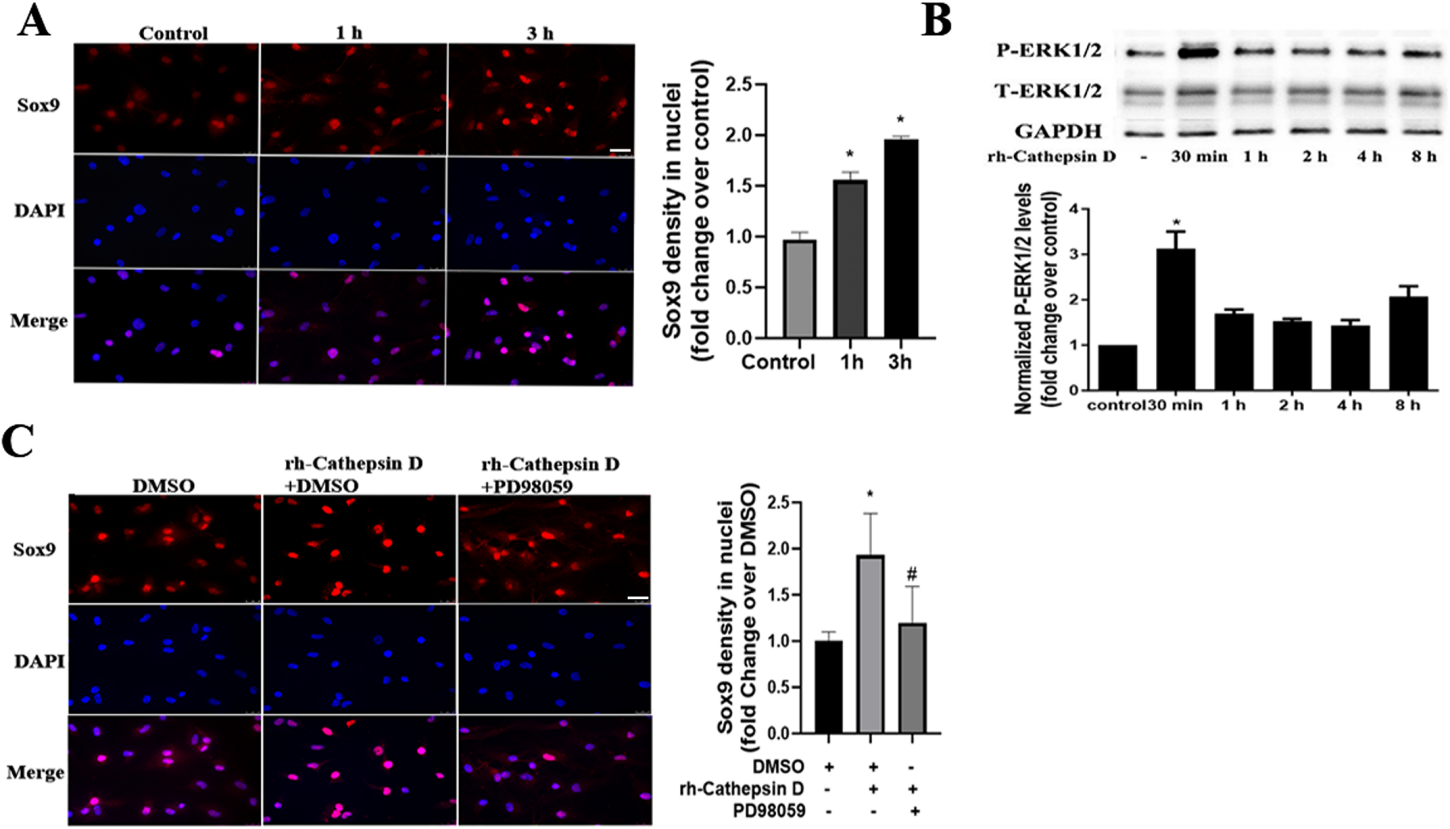
Cathepsin D activates the ERK1/2-Sox9 pathway in human AVICs. **(A)** Human AVICs from normal valves were treated with rh-cathepsin D (40 nM) for 1 or 3 h. Representative immunofluorescence images (original magnification 40× objective, scale bar = 100 µm) show that cathepsin D increases Sox9 (red) nuclear density. 4′, 6-Diamidino-2-phenylindole (DAPI, blue) was used for nuclear counterstaining. All data are presented as mean ± SE of 3 experiments using cell isolates from distinct valves. **(B)** Human AVICs from normal valves were treated with rh-cathepsin D (40 nM) for 30 min to 8 h. Representative immunoblots and densitometric data show that cathepsin D induces ERK1/2 phosphorylation. All data are presented as mean ± SE of 4 experiments using different cell isolates from distinct valves. *P < 0.05 vs. control. **(C)** AVICs of normal human valves were pretreated with specific ERK1/2 inhibitor (PD98059, 25 µM) 1 h before rh-cathepsin D treatment for 3 h. Representative immunofluorescence images (original magnification 40× objective, scale bar = 100 µm) show that inhibition of ERK1/2 suppresses the up-regulation of Sox9 nuclear density. Data are presented as mean ± SE of 3 experiments using cell isolates from distinct valves. *P < 0.05 vs. DMSO control. #*P *< 0.05 vs. rh-cathepsin D + DMSO.

Our previous study found that Sox9 plays a role in mediating the high phosphate-induced osteogenic activity in human AVICs (17). In the current study, we observed that treatment of human AVICs with rh-cathepsin D (40 nM) for 3 h increased the nuclear intensity of Sox9 (Figure 3A).

It is reported that the upregulation of the chondrogenic Sox9 gene by fibroblast growth factors is mediated by the MAPK pathway (21). To determine whether the ERK1/2 pathway is involved in the cathepsin D-induced Sox9 activation, we examined ERK1/2 phosphorylation after treatment with rh-cathepsin D. As shown in Figure 3B, ERK1/2 activation was evident at 30 min and preceded the increase in Sox9 density in the nuclei. Furthermore, ERK1/2 inhibition with PD98059 attenuated the elevation of Sox9 nuclear density in AVICs exposed to cathepsin D (Figure 3C).

**Figure 4:**
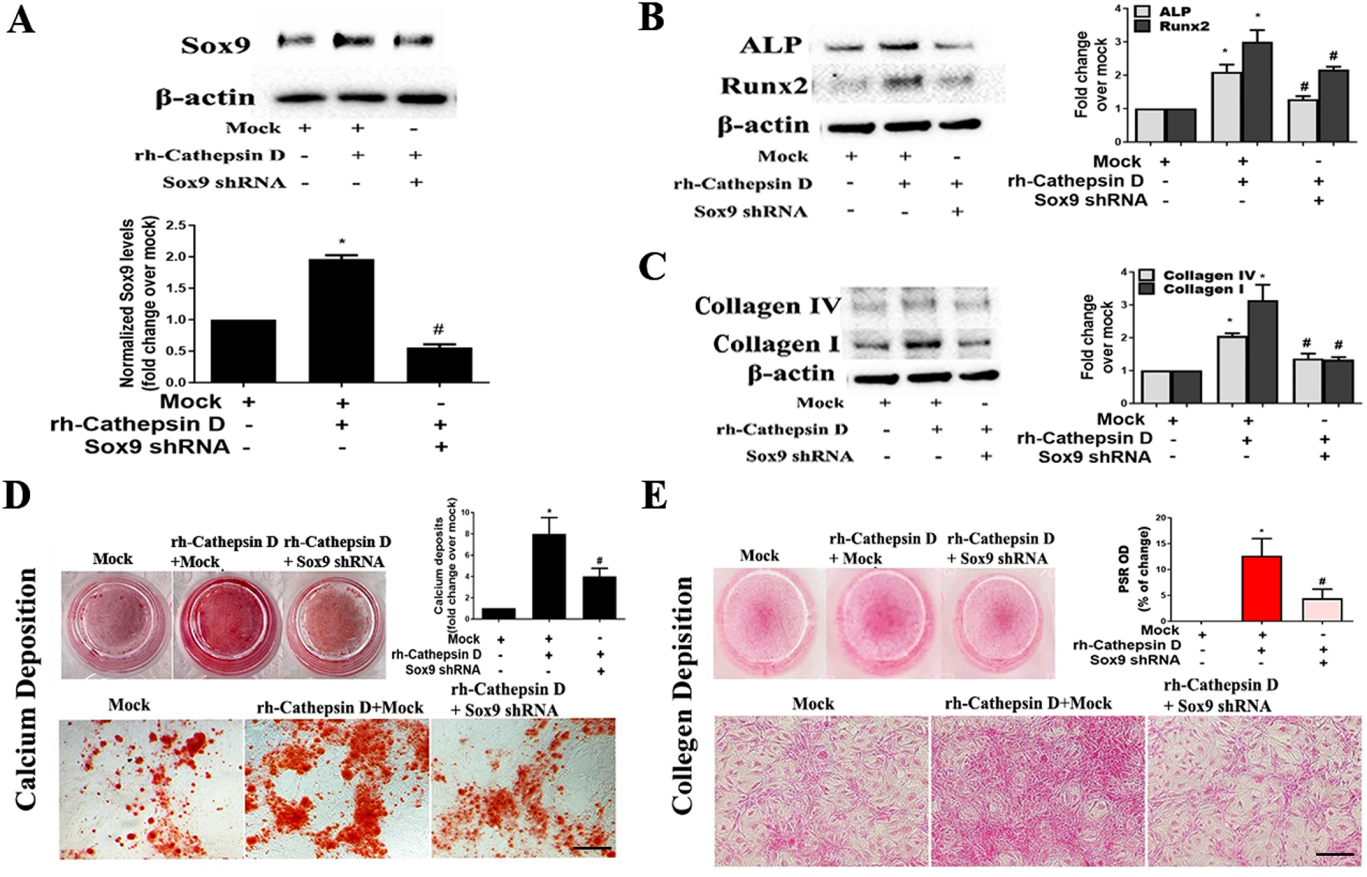
Knockdown of Sox9 attenuates the effect of cathepsin D on AVIC fibrocalcific activity. Human AVICs from 5 normal valves were pretreated with lentivirus expressing Sox9 shRNA (100 nmol/L) for 72 h, and then treated with rh-cathepsin D (40 nM). **(A)** Representative immunoblots and densitometric data show that knockdown of Sox9 with shRNA reduces Sox9 expression in cells exposed to rh-cathepsin D. **(B,C)** knockdown of Sox9 attenuates cathepsin D-induced expression of ALP, Runx2, collagen IV and collagen I. **(D)** Representative images of alizarin red staining and spectrophotometric data show that knockdown of Sox9 suppresses cathepsin D-induced calcium deposit formation in cells treated with rh-cathepsin D in conditioning medium for 10 days (10× objective for original magnification of the higher power images, scale bar = 100 µm). **(E)** PSR staining images and spectrophotometric data show that knockdown of Sox9 suppresses cathepsin D-induced collagen deposition in cells exposed to rh-cathepsin D for 14 days (10× objective for original magnification of the higher power images, scale bar = 100 µm). All data are presented as mean ± SE of 5 experiments using different cell isolates from distinct valves. *P < 0.05 vs. Mock. #*P *< 0.05 vs. rh-cathepsin D + Mock.

**Figure 5:**
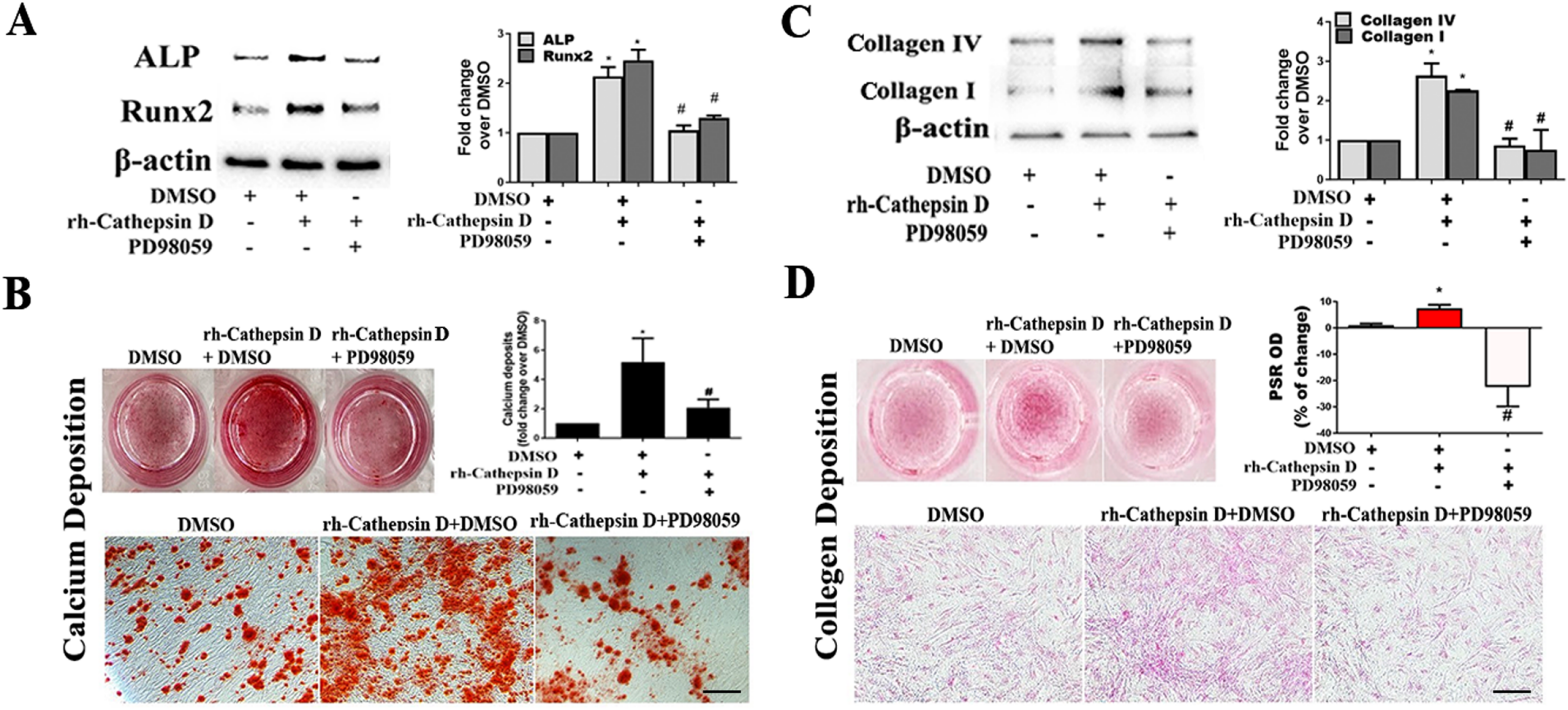
Inhibition of ERK1/2 suppresses AVIC fibrocalcific response to cathepsin D. **(A)** AVICs of 5 normal human valves were treated in triplicates with specific ERK1/2 inhibitor (PD98059, 25 µM) 1 h before rh-cathepsin D (40 nM) treatment. Representative immunoblots and densitometric data show that inhibition of ERK1/2 suppresses cathepsin D-induced ALP and Runx2 expression. **(B)** Representative images of alizarin red staining and spectrophotometric data show that inhibition of ERK1/2 reduces calcium deposit formation in AVICs with prolonged exposure to cathepsin D (10× objective for original magnification of the higher power images, scale bar = 100 µm). **(C)** Representative immunoblots and densitometric data show that inhibition of ERK1/2 suppresses cathepsin D-induced collagen IV and I expression. **(D)** PSR staining images and spectrophotometric data show that inhibition of ERK1/2 reduces the collagen deposition induced by prolonged cathepsin D exposure (10× objective for original magnification of the higher power images, scale bar = 100 µm). All data are presented as mean ± SE of 5 experiments using cell isolates from distinct valves. *P < 0.05 vs. DMSO. #*P *< 0.05 vs. rh-cathepsin D + DMSO.

To determine whether Sox9 mediates the up-regulation of fibrocalcific activity in human AVICs by cathepsin D, we applied specific shRNA to knockdown Sox9. As displayed in Figure 4A, treatment with Sox9 shRNA greatly reduced Sox9 protein levels in AVICs exposed to rh-cathepsin D. Importantly, knockdown of Sox9 attenuated rh-cathepsin D-induced expression of ALP, Runx2, collagen I and collagen IV in AVICs (Figures 4B,C). It also suppressed rh-cathepsin D-induced calcium and collagen deposition (Figures 4D,E). Since ERK1/2 is linked to Sox9 activation induced by cathepsin D, we tested the hypothesis that inhibition of ERK1/2 would attenuate cathepsin D-induced fibrocalcific response in human AVICs. We observed that inhibition of ERK1/2 essentially abrogated the effect of cathepsin D on the expression of ALP and Runx2 (Figure 5A) and calcium deposition (Figure 5B). It also suppressed cathepsin D-induced collagen expression (Figure 5C) and deposition (Figure 5D). Together, these results suggest that cathepsin D utilizes the ERK1/2-Sox9 pathway to upregulate the osteogenic and fibrogenic activities in human AVICs.

### 3.4 Soluble matrilin 2 up-regulates cathepsin D to elevate the osteogenic and fibrogenic activities in human AVICs

**Figure 6:**
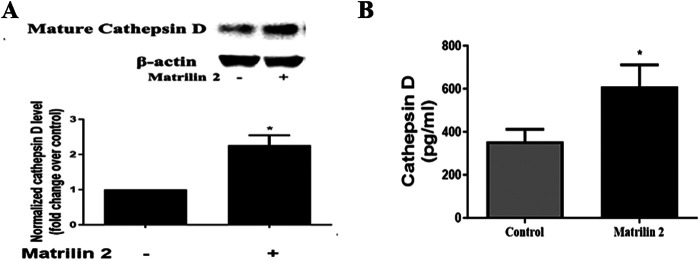
Matrilin 2 promotes the expression and release of cathepsin D in human AVICs. Normal AVICs from 5 distinct valves were treated in triplicates with matrilin 2 (2.0 μg/ml) for 48 h. **(A)** Cell lysates were collected to determine the level of cell-associated cathepsin D by immunoblotting. Representative immunoblots and densitometric data show that matrilin 2 up-regulates cathepsin D level. **(B)** Cell culture mediums were collected to determine the level of released pro-form cathepsin D using ELISA. The data shows that matrilin 2 promotes the release of cathepsin D. All data are presented as mean ± SE of 5 experiments using different cell isolates from distinct valves. *P < 0.05 vs. control.

Soluble matrilin 2 is pro-fibrocalcific to human AVICs (13). To determine whether matrilin 2 up-regulates the production of cathepsin D, we treated normal AVICs with soluble matrilin 2 (2.0 μg/ml) for 48 h. Cathepsin D levels in cell lysate and cell culture medium were analyzed by immunoblotting and ELISA, respectively. Figure 6A shows that treatment of normal AVICs with matrilin 2 increased the levels of cell-associated cathepsin D by two folds. Treatment with matrilin 2 also enhanced the release of cathepsin D by AVICs (609.6 ± 66.3 pg/ml vs. 355.2 ± 56.53 pg/ml in control; Figure 6B).

**Figure 7:**
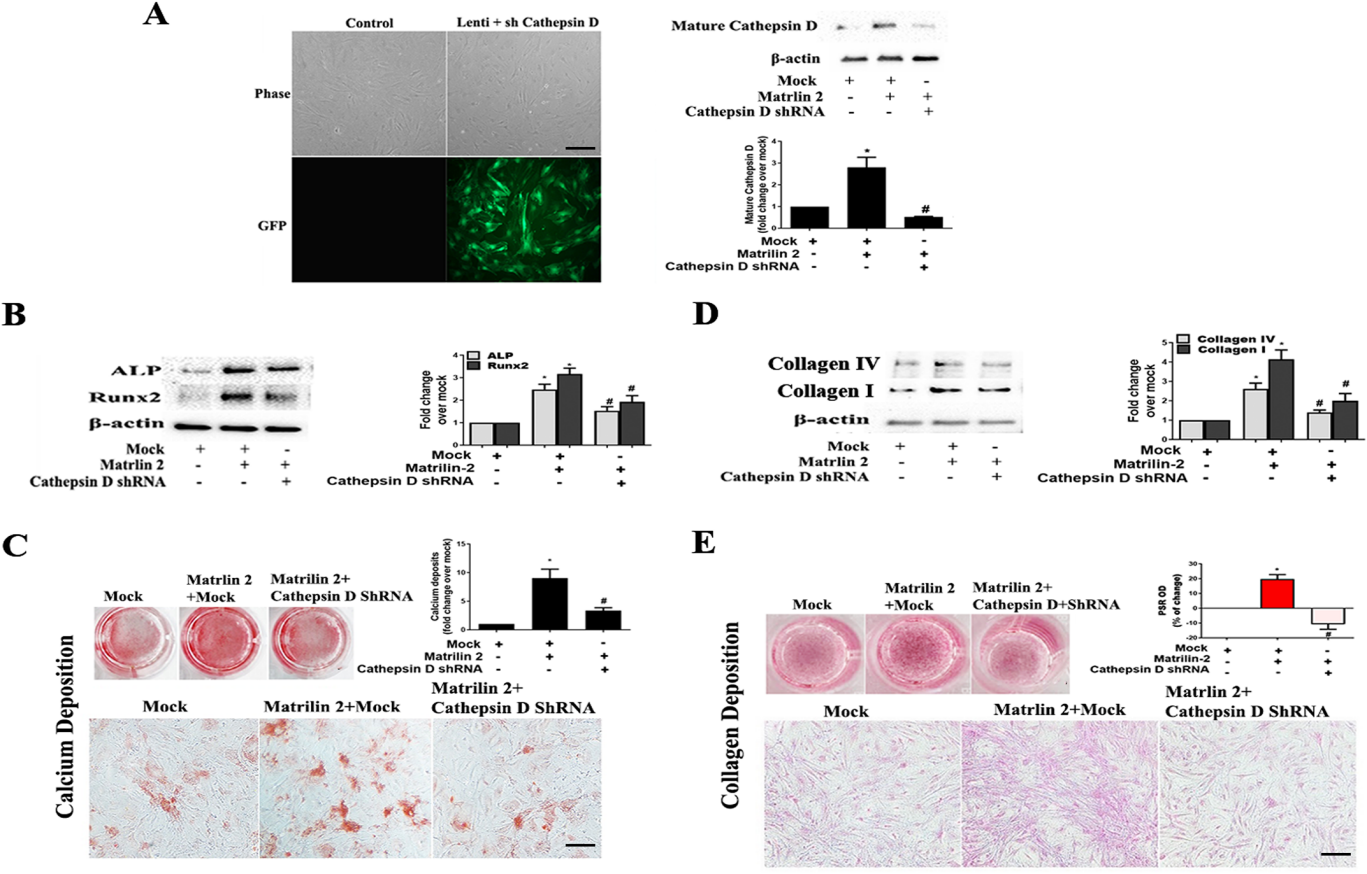
Cathepsin D is involved in the induction of fibrocalcific response by matrilin 2 in human AVICs. Human AVICs from 5 normal valves were pretreated with lentivirus expressing cathepsin D shRNA (100 nmol/L) for 72 h, and then exposed to matrilin 2 (2 μg/ml) for varied time (48 h–14 days). **(A)** GFP outlines cells that express cathepsin D shRNA (scale bar = 100 μm). Representative immunoblots and densitometric data show that knockdown of cathepsin D with shRNA reduced cathepsin D expression in AVICs exposed to matrilin 2 for 48 h. **(B,C)** Knockdown of cathepsin D attenuates ALP and Runx2 expression at 48 h of matrilin 2 exposure and calcium deposition in cells exposed to matrilin 2 for 10 days (10× objective for original magnification of the higher power images, scale bar = 100 µm). **(D,E)** Knockdown of cathepsin D attenuates collagen expression at 48 h of matrilin 2 exposure and collagen deposition in cells exposed to matrilin 2 for 14 days (10× objective for original magnification of the higher power images, scale bar = 100 µm). All data are presented as mean ± SE of 5 experiments using cell isolates from distinct valves. *P < 0.05 vs. Mock. #*P *< 0.05 vs. Matrilin 2 +Mock.

To determine whether cathepsin D contributes to the mechanism underlying matrilin 2-induced osteogenic and fibrogenic responses in human AVICs, we applied cathepsin D shRNA to knockdown its expression. As shown in Figure 7A, treatment with cathepsin D shRNA effectively suppressed cathepsin D expression. Knockdown of cathepsin D markedly reduced ALP and Runx2 expression and calcium deposition in cells exposed to matrilin 2 (Figures 7B,C). Knockdown of cathepsin D also reduced the levels of collagen I, collagen IV (Figure 7D), and collagen deposition (Figure 7E) in AVICs exposed to matrilin 2. Therefore, cathepsin D is involved in the induction of fibrocalcific response by matrilin 2 in human AVICs.

## 4 Discussion

To develop pharmacological treatments for prevention of CAVD progression, it is important to understand the mechanisms underlying the progression of this disease. In the present study, we discovered that AVICs from aortic valves affected by CAVD produce and release greater levels of cathepsin D than AVICs from normal aortic valves, and that cathepsin D elevates the osteogenic and fibrogenic activities in human AVICs through the ERK1/2-Sox9 signaling pathway. In addition, the production and release of cathepsin D in human AVICs can be enhanced by soluble ECM protein matrilin 2, and cathepsin D mediates matrilin 2-induced fibrocalcific response in human AVICs. Our findings suggest that cathepsin D functions as a novel pro-fibrocalcific mediator in human aortic valves and may play a role in the mechanism underlying CAVD progression.

By using proteomic analysis, we obtained protein profiles of AVICs from normal and calcified aortic valves. We found that 64 proteins are upregulated in AVICs from aortic valves affected by CAVD compared to AVICs from normal valves. Among those proteins, cathepsin D level increased by 1.8 folds in AVICs of diseased valves. Immunoblotting confirmed that AVICs from diseased valves have higher levels of cell-associated cathepsin D. In addition, immunoblotting and ELISA analyses demonstrate that cells from diseased aortic valves secrete greater levels of cathepsin D. The present study found that over-production of cathepsin D is a characteristic change in AVIC pathobiology associated with CAVD. Interestingly, our previous study utilized microarray analysis to investigate mRNA expression changes between normal and diseased AVICs (18). With the fold change cutoff at 1.8, we did not observe a significant alteration in the expression of cathepsin D at the mRNA level. This discrepancy could potentially be attributed to a higher translation rate of the cathepsin D mRNA, as well as a lower degradation or higher stability rate of the cathepsin D protein in AVICs derived from diseased aortic valves.

Cathepsin D is present in most mammal cells and tissues, including valvular interstitial cells (22). They are known for their key role in protein turnover and actively contribute to ECM remodeling (23). Unlike other members of the aspartic protease family that are mostly secretory, cathepsin D is stored in the cytoplasm and lysosomes under physiological conditions (20, 24). However, several studies indicate that cathepsin D is released from cells under certain pathological conditions and is involved in the pathobiology of several diseases (24–26). In this regard, studies in neuronal cells have shown that cathepsin D is released and is capable of provoking the Bax-dependent mechanism of apoptosis (27). Cathepsin D is also found in macrophage-rich regions of atherosclerotic lesions (28), indicating a role of increased cathepsin D levels in atherosclerotic plaque formation and enlargement. Patients with chronic kidney disease (CKD) have higher levels of cathepsin D in circulation, and cathepsin D promotes the progression of renal fibrosis (29). CKD, atherosclerosis and CAVD share common risk factors. Over-production of cathepsin D may be one of factors in the pathobiology of these diseases. Our study found rh-cathepsin D elevates the fibrogenic and osteogenic activities in human AVICs. This is reflected by over-expression of fibrogenic and osteogenic mediators, as well as greater deposition of collagen and calcium, as indicators of *in vitro* fibrogenic and osteogenic activities. Our study highlights the novel role of cathepsin D in up-regulating AVIC fibrogenic and osteogenic activities associated CAVD pathogenesis. The novel findings of the present study support the notion that higher cathepsin D levels in AVICs promote CAVD progression.

Cathepsin D production and release can be modulated by pro-inflammatory mediators, such as cytokines and chemokines, in different cell types (30, 31). In this regard, pro-inflammatory stimuli, including lipopolysaccharide (LPS, a TLR4 agonist) and interferon-γ, up-regulate the expression of cathepsin D that subsequently enhances the inflammatory activity in macrophages (30). The TLR3 agonist, poly IC, is found to induce cathepsin D release from lysosomes and subsequent NF-κB activation in dendritic cells (31). As higher TLR activity is present in diseased aortic valves, and activation of TLR up-regulates AVIC fibrogenic and osteogenic activities (15), it is likely that pro-inflammatory mediators exist in aortic valves with chronic inflammation up-regulate the expression of cathepsin D in AVICs and that cathepsin D plays a role in mediating AVIC fibrocalcific activation by pro-inflammatory mechanisms.

Previous studies demonstrate that soluble ECM proteins, including biglycan (32) and matrilin 2 (14), elicit the pro-inflammatory activity in human AVICs. Our previous study found matrilin 2 accumulation in diseased aortic valves, and soluble matrilin 2 functions as a danger-associated molecular pattern (DAMP) to activate TLR2/4 in human AVICs, leading to the release of pro-inflammatory cytokines (14). More importantly, soluble matrilin 2 is potent in elevation of osteogenic activities in human AVICs through a TLR2/4-dependent mechanism (13). In the present study, we observed that soluble matrilin 2 has a pro-fibrogenic effect on human AVICs. As cathepsin D up-regulates fibrogenic activities in AVICs, we tested the hypothesis that soluble matrilin 2 induces cathepsin D to up-regulate AVIC fibrogenic and osteogenic activities. Our data show that exposure of human AVICs to soluble matrilin 2 markedly increased the levels of both cell-associated cathepsin D and secreted cathepsin D. Interestingly, knockdown of cathepsin D in AVICs markedly reduced the pro-fibrocalcific effect of soluble matrilin 2, resulting in attenuated deposition of collagen and calcium by those cells. Together with the observation that cathepsin D is potent in upregulating the fibrogenic and osteogenic activities in human AVICs, the findings from the experiments exploring the matrilin 2-cathepsin D-fibrocalcification cascade highlight the important role of cathepsin D in the inflammatory mechanism of aortic valve fibrocalcification. Targeting the inflammatory pathways that modulate cathepsin D expression and activity may have therapeutic potential for prevention of CAVD progression.

Sox9 is an SRY-related transcription factor transcriptional factor of chondrogenesis. It modulates the expression and distribution of valvular ECM proteins (33) and regulates bone mineralization in the skeletal system (34). Previous study found that Sox9 was overexpressed in the activated hepatic stellate cells that are responsible for the production of type 1 collagen to promote liver fibrosis (34). Sox9 also regulates the production of osteopontin, a biomarker for osteogenic activity (34). Our previous study found that a higher level of phosphate upregulated the expression of Sox9 and enhances osteogenic activity in human AVICs (17). In addition, ectopic Sox9 expression occurs during the activation of fibrogenic cells from the human adult liver and mediated the expression of the major component of fibrotic ECM, including type 1 and type 2 collagen (35). It is likely that Sox9 plays a role in cellular osteogenesis and fibrogenesis. In the current study, we observed that cathepsin D has the capacity of activating Sox9 and thus up-regulates the fibrogenic and osteogenic activities in human AVICs. Interestingly, the MAPK pathway has been reported to be required in the FGF-induced expression and activation of Sox9 in chondrocytes (21), as well as in undifferentiated mesenchymal cells (36). Previous studies found that ERK1/2 signaling is involved in aortic valve fibrosis and calcification. The present study found that cathepsin D induces rapid activation of ERK1/2 followed by an increase in Sox9 density in the nuclei. The greater nuclear density of Sox9 in AVICs exposed to cathepsin D appears to involve intranuclear translocation and elevated nuclear expression of this transcription factor as its cytoplasmic level decreases, and its protein level is upregulated by cathepsin D in AVICs. Further, inhibition of ERK1/2 attenuates Sox9 accumulation in the nucleus, indicating a link of ERK1/2 signaling to increased Sox9 activity in the nucleus of human AVICs exposed to cathepsin D. Interestingly, inhibition of either ERK1/2 or Sox9 reduces the potency of cathepsin D in elevating the fibrocalcification in human AVICs. Thus, the ERK1/2-Sox9 signaling pathway plays a critical role in mediating the up-regulation of AVIC fibrogenic and osteogenic activities by cathepsin D. This signaling cascade could be a potential target for suppression of AVIC pathobiology associated with CAVD progression.

The current study identified cathepsin D as a mediator of AVIC fibrogenic and calcific activities, critical pathobiology in CAVD progression. It should be pointed out, however, that focusing on one factor is one of the limitations of this study. Proteomic analysis found 340 proteins present in AVICs from diseased valves while they were not detectable in AVICs of normal valves. Additional 64 proteins were upregulated in AVICs from diseased valves in comparison to AVICs from normal valves. It is possible that some of these induced and up-regulated proteins also play a role in elevating the fibrogenic and calcific activities in AVICs of diseased aortic valves. Furthermore, it is also possible that up-regulation of pro-fibrocalcific factors and down-regulation of anti-fibrocalcific factors in concert to elevate AVIC fibrogenic and calcific activities involved CAVD progression. Further studies are needed to elaborate the molecular interaction of Cathepsin D with other factors in the mechanism aortic valve fibrocalcification.

## 5 Conclusions

In the present study, we discovered that AVICs of diseased aortic valves express and release greater levels of cathepsin D. Cathepsin D activates the ERK1/2-Sox9 signaling pathway to up-regulate fibrogenic and osteogenic activities in human AVICs. Pro-inflammatory stimulus soluble matrilin 2 is capable of inducing cathepsin D expression in human AVICs, and cathepsin D has a mechanistic role in mediating the pro-fibrocalcific effect of soluble matrilin 2. Pharmaceutical agents that antagonize cathepsin D or inhibit the ERK1/2-Sox9 signaling pathway might have the potential to suppress valvular fibrocalcific activity involved in the pathobiology of CAVD.

## Data Availability

The original contributions presented in the study are publicly available. This data can be found here: https://github.com/cathepsinD/cathepsinD_dataset.
