# Supplementary material for: Cathepsin D elevates the fibrocalcific activity in human aortic valve cells through the ERK1/2-Sox9 pathway
Source: Front Cardiovasc Med. 2024 Sep 24;11:1410862. doi: 10.3389/fcvm.2024.1410862 (PMC11458440; doi:10.3389/fcvm.2024.1410862)

Supplementary materials

Supplemental Table 1. Valve donor demographics

Group	Age	Gender	Diagnosis
N1	58	Female	Idiopathic Dilated Cardiomyopathy
N2	52	Female	Ischemic Cardiomyopathy
N3	57	Male	Idiopathic Dilated Cardiomyopathy
N4	60	Male	Ischemic Cardiomyopathy
N5	62	Male	Ischemic Cardiomyopathy
D1	59	Male	CAVD
D2	62	Male	CAVD
D3	62	Female	CAVD
D4	51	Male	CAVD
D5	64	Male	CAVD

N=normal aortic valve; D=diseased aortic valve

Supplemental Table 2. Upregulated and downregulated proteins in the interstitial cells of calcified aortic valves

Gene	Identified Proteins	Rel Quant (Fold)
TFG_HUMAN	Protein TFG	2.5
HSPB1_HUMAN	Heat shock protein beta-1	2.4
BASP1_HUMAN	Brain acid soluble protein 1	2.2
K22E_HUMAN	Keratin, type II cytoskeletal 2 epidermal	2.2
TCPB_HUMAN	T-complex protein 1 subunit beta	2.1
S10AA_HUMAN	Protein S100-A10	2.0
EF1A1_HUMAN	Elongation factor 1-alpha 1	2.0
LKHA4_HUMAN	Leukotriene A-4 hydrolase	2.0
ATPO_HUMAN	ATP synthase subunit O, mitochondrial	1.9
HNRPU_HUMAN	Heterogeneous nuclear ribonucleoprotein U	1.9
NCKP1_HUMAN	Nck-associated protein 1	1.9
K1C10_HUMAN	Keratin, type I cytoskeletal 10	1.8
KHDR1_HUMAN	KH domain-containing, RNA-binding, signal transduction-associated protein 1	1.8
CATD_HUMAN	Cathepsin D	1.8
GDIR1_HUMAN	Rho GDP-dissociation inhibitor 1	1.8
PGK1_HUMAN	Phosphoglycerate kinase 1	1.7
HYEP_HUMAN	Epoxide hydrolase 1	1.7
FKB1A_HUMAN	Peptidyl-prolyl cis-trans isomerase FKBP1A	1.7
DDAH1_HUMAN	N(G),N(G)-dimethylarginine dimethylaminohydrolase 1	1.7
FLNB_HUMAN	Filamin-B	1.7
ACBP_HUMAN	Acyl-CoA-binding protein	1.7
AHNK2_HUMAN	Protein AHNAK2	1.7
THIM_HUMAN	3-ketoacyl-CoA thiolase, mitochondrial	1.7
TKT_HUMAN	Transketolase	1.7
MTPN_HUMAN	Myotrophin	1.7
DBNL_HUMAN	Drebrin-like protein	1.7
GSTO1_HUMAN	Glutathione S-transferase omega-1	1.6
PIMT_HUMAN	Protein-L-isoaspartate(D-aspartate) O-methyltransferase	1.6
XPO1_HUMAN	Exportin-1	1.6
BST1_HUMAN	ADP-ribosyl cyclase/cyclic ADP-ribose hydrolase 2	1.6
LDHA_HUMAN	L-lactate dehydrogenase A chain	1.6
ALDH2_HUMAN	Aldehyde dehydrogenase, mitochondrial	1.6
TAGL2_HUMAN	Transgelin-2	1.6
LIMA1_HUMAN	LIM domain and actin-binding protein 1	1.6
COMT_HUMAN	Catechol O-methyltransferase	1.6
CH60_HUMAN	60 kDa heat shock protein, mitochondrial	1.6
TRXR1_HUMAN	Thioredoxin reductase 1, cytoplasmic	1.6
PLOD2_HUMAN	Procollagen-lysine,2-oxoglutarate 5-dioxygenase 2	1.6
MYOF_HUMAN	Myoferlin	1.6
SERPH_HUMAN	Serpin H1	1.6
HDGF_HUMAN	Hepatoma-derived growth factor	1.6
RCN3_HUMAN	Reticulocalbin-3	1.6

DPY30_HUMAN	Protein dpy-30 homolog	1.6
S10AG_HUMAN	Protein S100-A16	1.6
UBP2L_HUMAN	Ubiquitin-associated protein 2-like	1.6
IQGA1_HUMAN	Ras GTPase-activating-like protein IQGAP1	1.6
ZN649_HUMAN	Zinc finger protein 649	1.6
COR1C_HUMAN	Coronin-1C	1.6
STX7_HUMAN	Syntaxin-7	1.6
UBA1_HUMAN	Ubiquitin-like modifier-activating enzyme 1	1.5
MAP4_HUMAN	Microtubule-associated protein 4	1.5
VINC_HUMAN	Vinculin	1.5
FBLN3_HUMAN	EGF-containing fibulin-like extracellular matrix protein 1	1.5
UB2V1_HUMAN	Ubiquitin-conjugating enzyme E2 variant 1	1.5
ICAL_HUMAN	Calpastatin	1.5
PRKDC_HUMAN	DNA-dependent protein kinase catalytic subunit	1.5
GBB1_HUMAN	Guanine nucleotide-binding protein G(I)/G(S)/G(T) subunit beta-1	1.5
GBB2_HUMAN	Guanine nucleotide-binding protein G(I)/G(S)/G(T) subunit beta-2	1.5
ANXA2_HUMAN	Annexin A2	1.5
ITA3_HUMAN	Integrin alpha-3	1.5
DHE3_HUMAN	Glutamate dehydrogenase 1, mitochondrial	1.5
IDHC_HUMAN	Isocitrate dehydrogenase [NADP] cytoplasmic	1.5
EHD2_HUMAN	EH domain-containing protein 2	1.5
OR1M1_HUMAN	Olfactory receptor 1M1	1.5
SDHB_HUMAN	Succinate dehydrogenase iron-sulfur subunit	0.6
CO1A2_HUMAN	Collagen alpha-2(I) chain	0.6
EF1B_HUMAN	Elongation factor 1-beta	0.5
WDR1_HUMAN	WD repeat-containing protein 1	0.5
AP0C3_HUMAN	Apolipoprotein C-III	0.4
GANAB_HUMAN	Neutral alpha-glucosidase AB	0.4
CATB_HUMAN	Cathepsin B	0.2

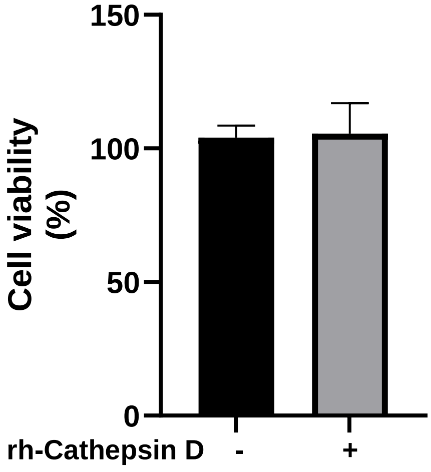

Supplementary Figure 1. Cathepsin D has no effect on AVIC viability. AVICs from normal valves were treated with rh-Cathepsin D for 48 h. CCK-8 assay was performed using an assay kit from Enzo Life Sciences International (Plymouth Meeting, PA, USA) following the manufacturer's protocol. In brief, cells were incubated with CCK-8 labeling reagent at 37 °C for 4 h. Formazan dye formation was evaluated by scanning with a spectrophotometer at 450 nm. The results of untreated controls are expressed as 100%. Treatment with cathepsin D had a minimal effect on cell viability.

Graphic Abstract

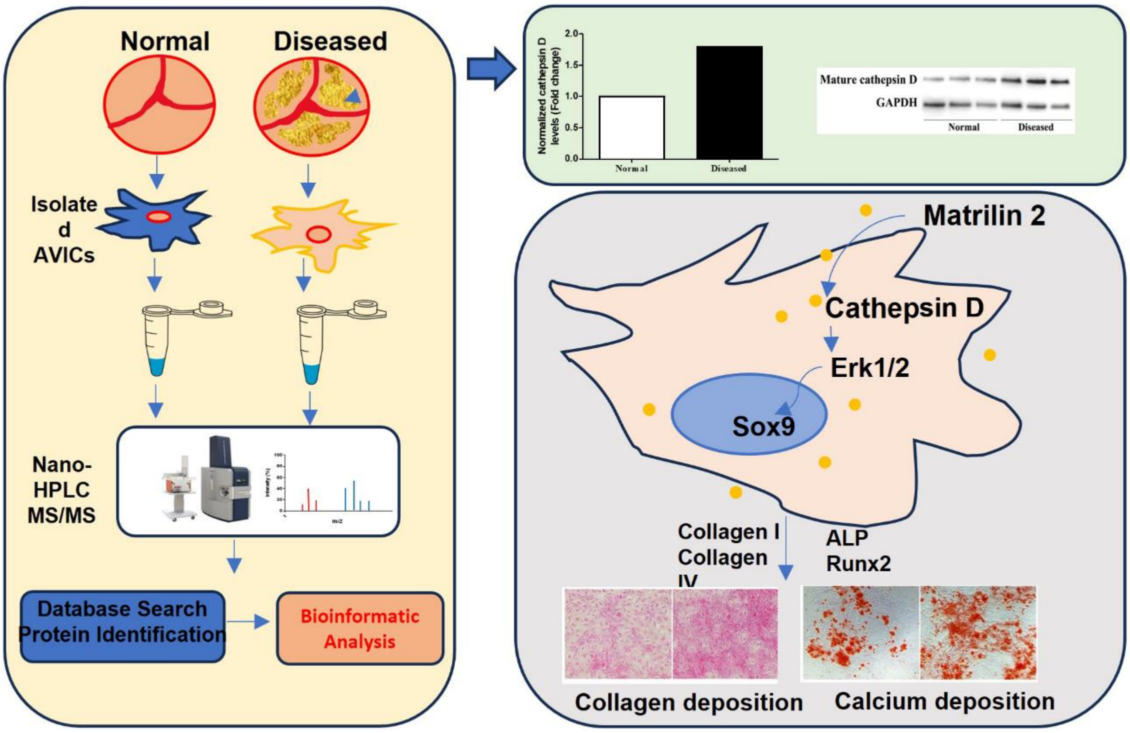

Supplement: Supplementary file 1 [file Datasheet1.pdf]
